# Ulnar Artery Aneurysm Presenting as Raynaud’s Phenomenon: A Case Report

**DOI:** 10.7759/cureus.83861

**Published:** 2025-05-10

**Authors:** Dasith K Jayawickrama, Joel Arudchelvam, Rohan Jayawardena, Kugapiragash Rajkumar

**Affiliations:** 1 University Vascular and Transplant Surgery Department, National Hospital of Sri Lanka, Colombo, LKA

**Keywords:** aneurysm excision, digital ischemia, hypothenar hammer syndrome, raynaud’s phenomenon, ulnar artery aneurysm

## Abstract

Ulnar artery aneurysm (UAA) is an uncommon cause of digital ischemia. It is most often encountered in patients with repetitive injuries to the hypothenar region. UAA can cause significant morbidity, such as pain, nerve compression, and even acute digital ischemia if it is not identified early and treated accordingly. We present a case of a professional hockey player who presented with signs of Raynaud’s phenomenon in the right-side ring finger, which was treated with UAA excision and primary end-to-end repair. A brief literature review of the literature is furthermore discussed.

## Introduction

Ulnar artery aneurysm (UAA), which was initially described by von Rosen in 1934, and in 1970, it was described as hypothenar hammer syndrome (HHS) by Conn et al. [1,2]. UAA occurs in patients with repetitive trauma to the superficial branch of the ulnar artery [3]. The superficial branch of the ulnar artery is prone to getting compressed against the hook of the hamate bone at the distal end of Guyon's canal, leading to aneurysm formation [4]. UAA has a striking male dominance, with a male-to-female ratio of 9:1, and in 75% of the cases, it involves the dominant hand [4]. Patients present with features of acute digital ischemia and/or ulnar neuropathy.

Raynaud's phenomenon is characterized by vasoconstriction of digital arteries and cutaneous arteries, leading to digital ischemia. This condition was initially described by Maurice Raynaud in 1862. Raynaud's phenomenon can be classified as primary and secondary depending on the etiological factors. Primary Raynaud's phenomenon is a diagnosis of exclusion if no etiological factors are found in the workup [5].

We report a case of UAA with acute digital ischemia in a professional athlete. It was treated with aneurysm excision and primary end-to-end repair of the ulnar artery.

## Case presentation

A 47-year-old male presented with right-sided ring finger coldness with paresthesia and bluish discoloration for a six-hour duration. The signs appeared after he performed a set of pushups. He also noticed a painless pulsatile lump over the hypothenar area for one year duration and had a feeling of a “hard ball rolling under skin” over the hypothenar area for the last three days while doing pushups. He had poorly controlled hypertension for 10 years. The dominant hand of the patient is the right side. There were no previous injuries or surgeries done in the right hand.

**Figure 1:**
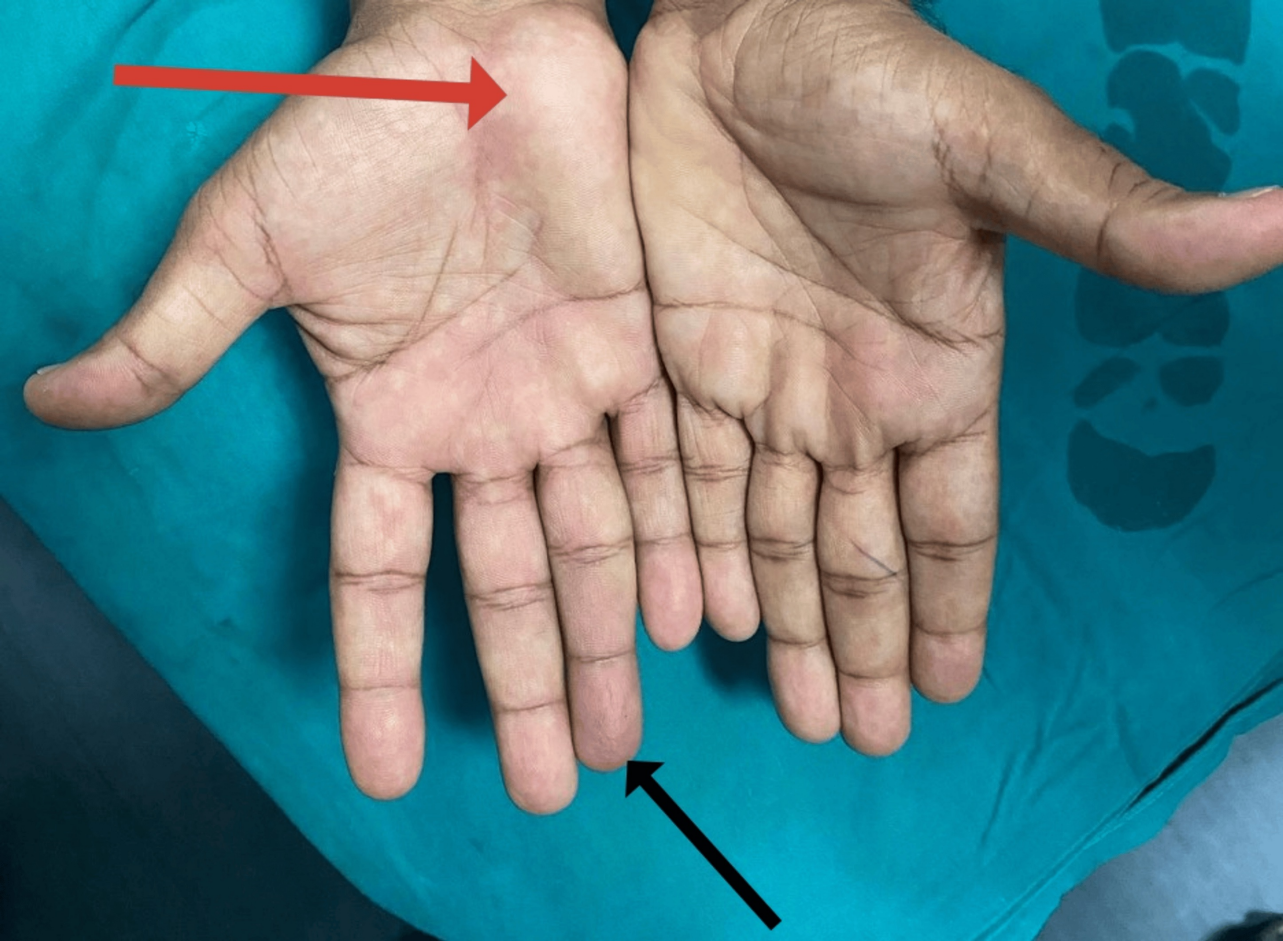
Right-side hypothenar region lump. Note the blueish discoloration of the tip of the ring finger (black arrow) and the right side hypothenar region discrepancy (red arrow).

On examination, a pulsatile lump was noticed over the right hypothenar region (Figure 1). There were no features of ulnar nerve neuropathy. The distal segment of the ring finger was bluish and cold. There was paresthesia of the ring finger. The strength of intrinsic muscles was preserved. Allen's test was done, and both arteries were vascularizing the hand.

**Figure 2:**
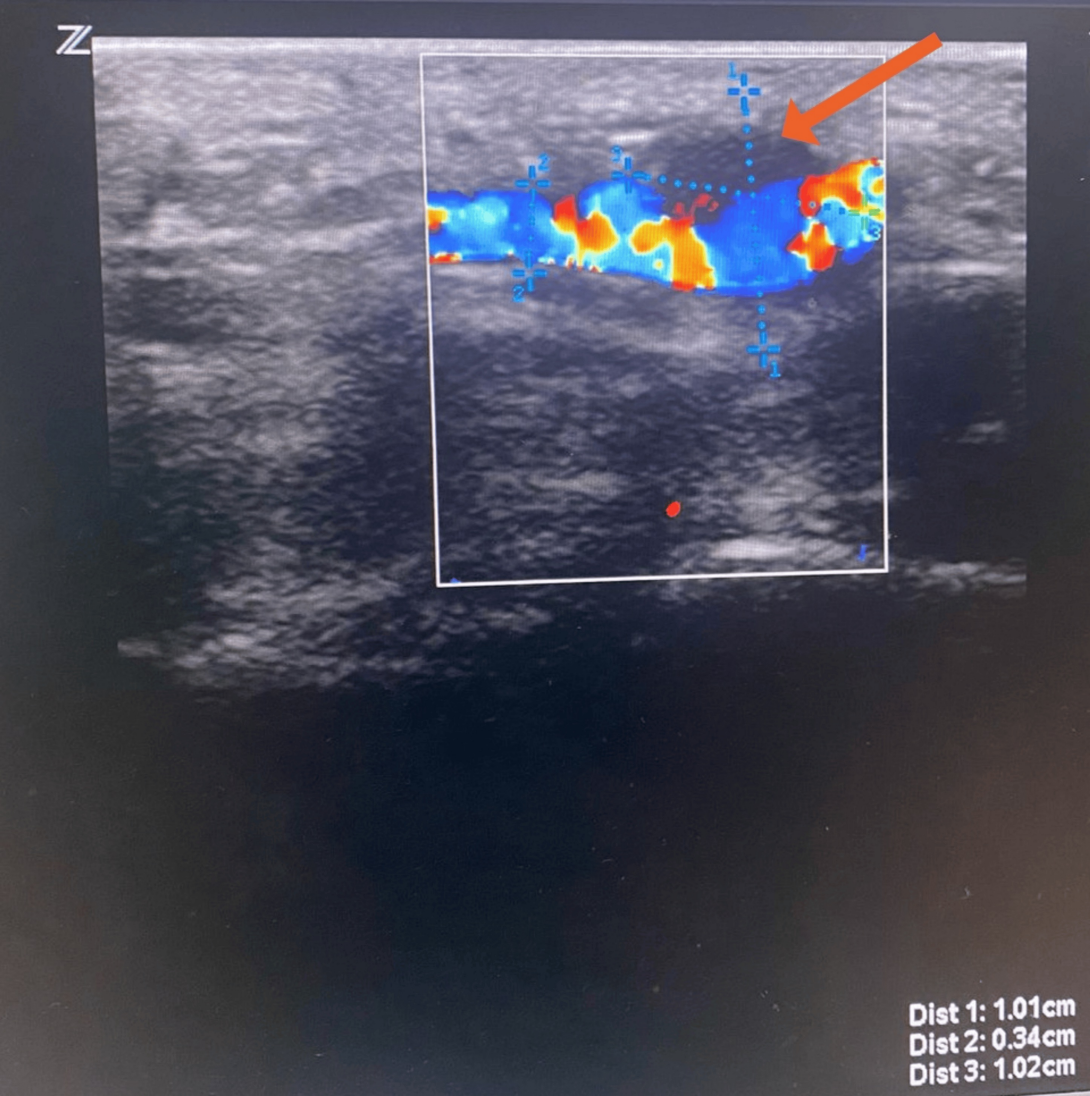
Arterial duplex scan showing ulnar artery aneurysm with thrombus (orange arrow).

The patient underwent an arterial duplex scan, which showed a 1 x 1 cm aneurysm of the ulnar artery (Figure 2). A thrombus was present within the aneurysm.

**Figure 3:**
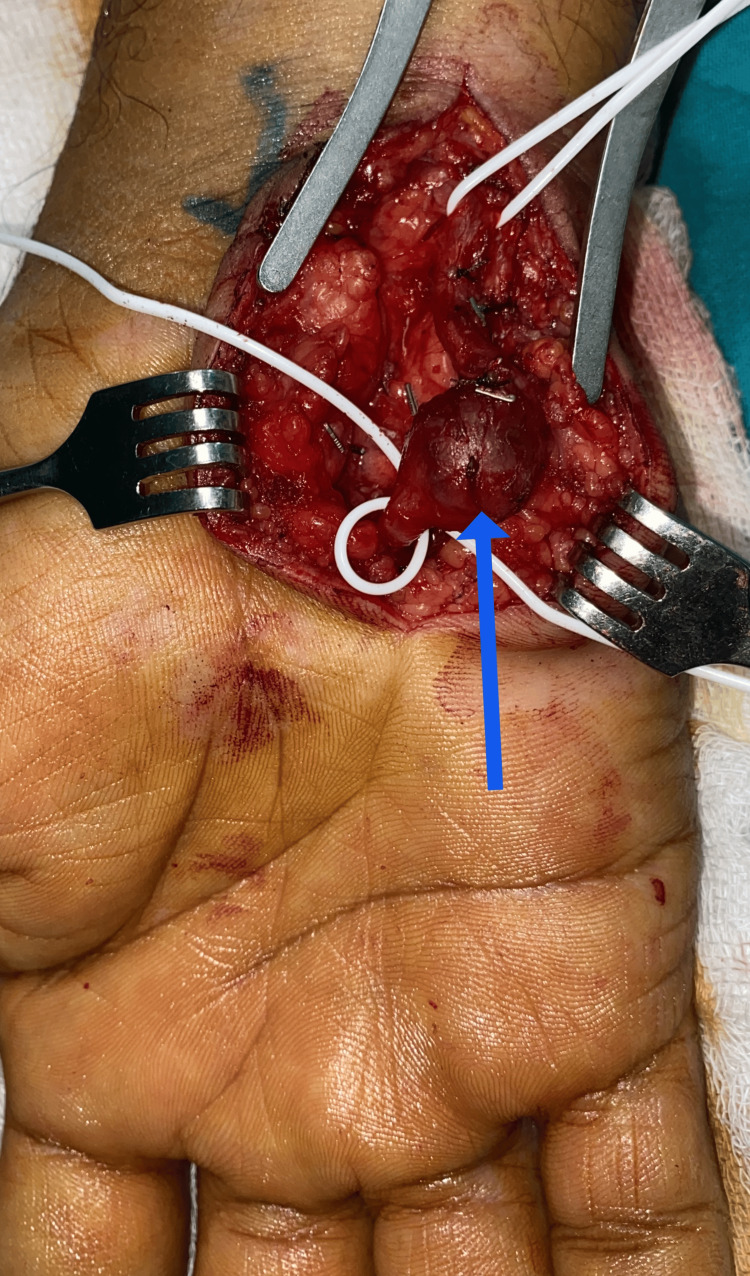
Ulnar artery aneurysm (blue arrow).

**Figure 4:**
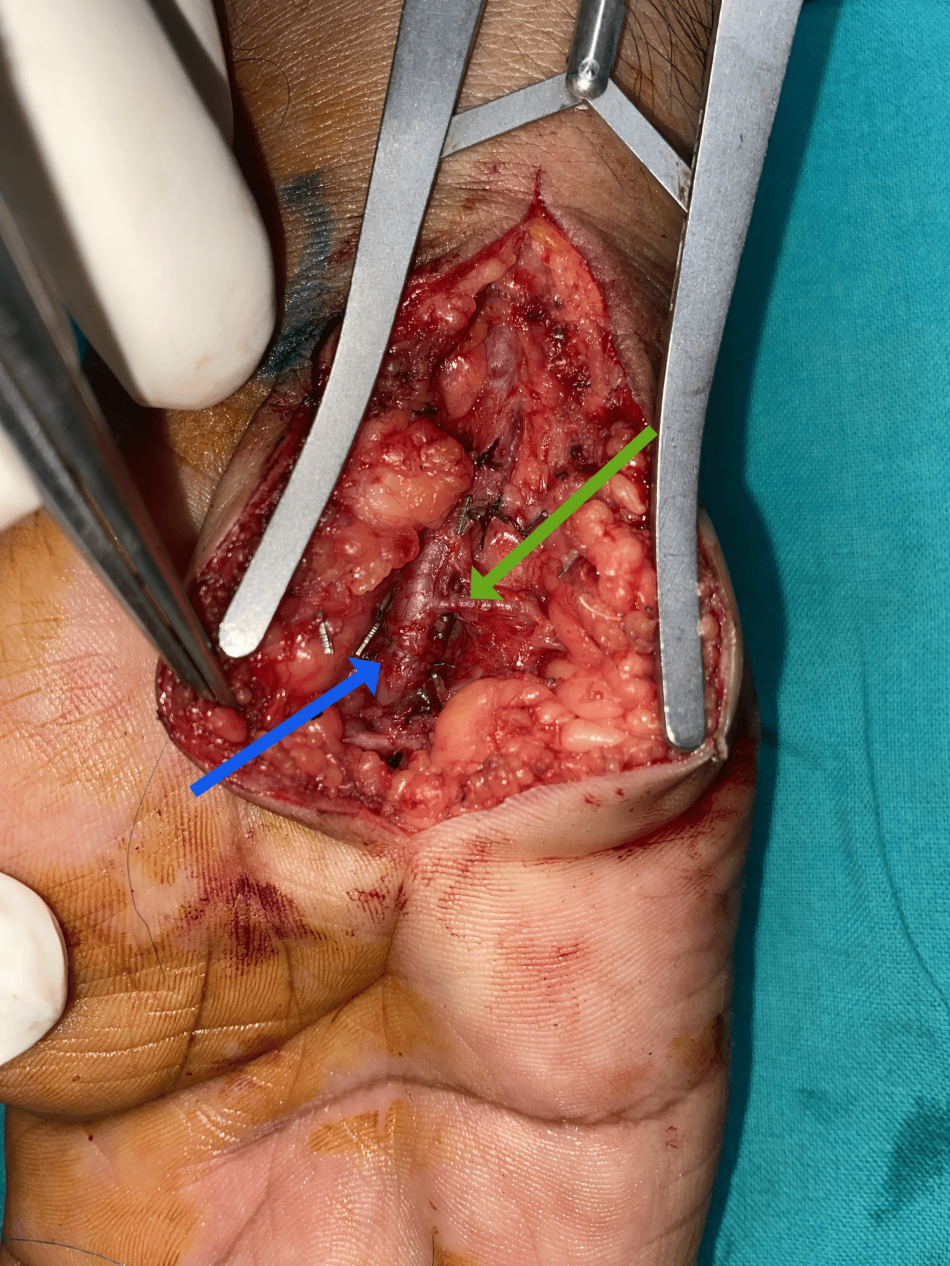
End-to-end anastomosis (blue arrow) and deep branch of the ulnar artery (green arrow).

**Figure 5:**
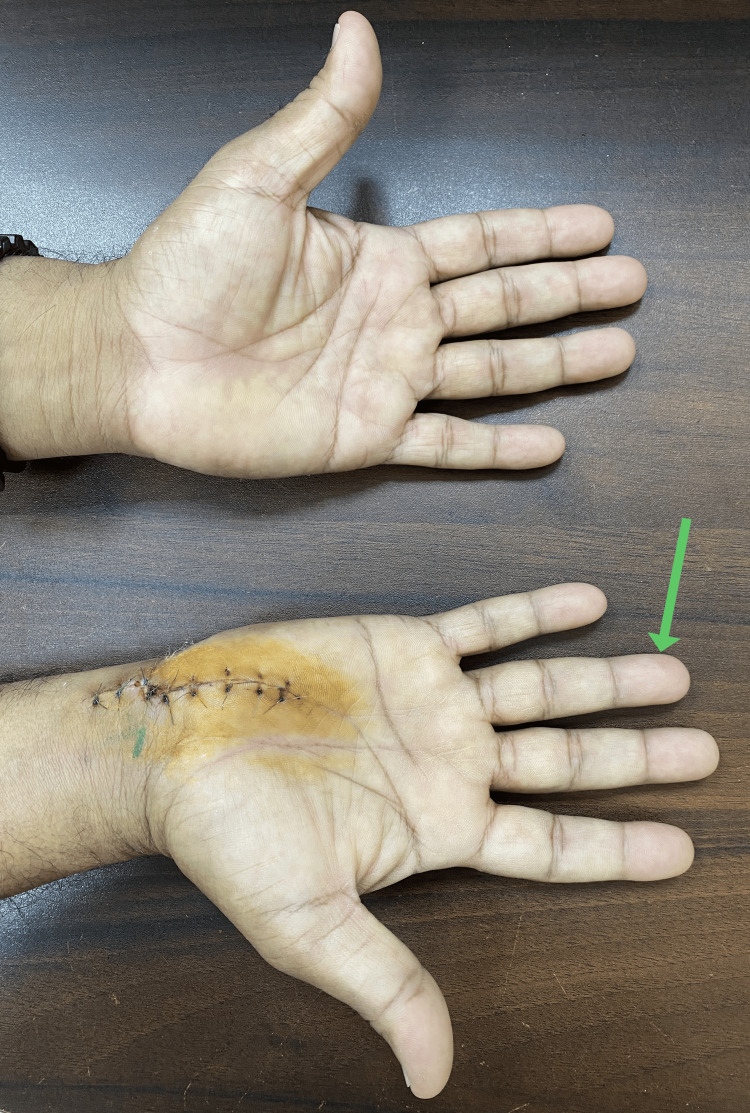
Two weeks post-operative follow-up. No residual color changes in the right ring finger (green arrow).

An ulnar artery aneurysm excision and repair were performed under regional anesthetic block. Intraoperatively, a fusiform aneurysm of the ulnar artery was noticed (Figure 3). The aneurysm was resected, and a primary end-to-end anastomosis of the ulnar artery was done with 8-0 Polypropylene sutures (Figure 4). The patient made an uneventful recovery and was discharged on oral aspirin 75 mg daily and statins. At two weeks post-operative follow-up, there were no residual color changes and no temperature difference. No signs of surgical site infections are noted (Figure 5).

## Discussion

UAA is an uncommon etiology for digital ischemia. This condition was initially described by von Rosen in 1934 [1], and the name hypothenar hammer syndrome was given by Conn et al. in 1970 [2]. It is commonly encountered in laborers who have repetitive and vibratory trauma to the hypothenar area, as the name suggests, due to using the hypothenar region as a hammer for impacting objects. It is also encountered in other professions such as athletes, mechanics, butchers, bikers, etc. [3]. A large cohort study including 1,300 workers demonstrated that the incidence of HHS among the participants was about 1.6% [3]. A characteristic male-dominant pattern is seen in HHS, with 75% involving the dominant hand [4].

It is believed that existing inherent arterial abnormalities and the compression of the ulnar artery by the hook of hamate cause the UAA formation [3,4]. Micro-emboli from the aneurysm can migrate distally and cause ischemia of the digital arteries, mimicking Raynaud's phenomenon [3-6].

Patients with UAA present with features of digital ischemia such as paresthesia, coldness, and discoloration. If the patients present with significant delay, they may even have dry gangrene of the fingers [7]. Since the ulnar nerve is near the ulnar artery, the compression exerted by the aneurysm can cause ulnar neuropathy [8].

History and examination findings may be adequate to come to a diagnosis. Allen’s test should be performed to check for the vascularization of the hand by the radial artery and to assess the arch insufficiency. To confirm UAA, a Doppler ultrasound scan could be used. Apart from a USS, computed tomographic (CT) angiogram, magnetic resonance (MR) angiogram, or digital subtraction arteriogram can be used to demonstrate the vascular anatomy, location, and size of the aneurysm [9].

Treatment of UAA depends on the severity of the symptoms and the presence of digital ischemia. Conservative treatment includes avoiding further trauma, analgesics, cessation of smoking, palmar padding, antiplatelets such as aspirin, and vasodilators such as calcium channel blockers.

Percutaneous catheter-guided embolectomy and surgical exploration of the aneurysm and excision, followed by reconstruction with end-to-end primary anastomosis or with an arterial or venous graft, are available as options for treatment [9,10]. Adjuvant treatment modalities include thoracic sympathectomy, intra-arterial vasodilators, and thrombolysis.

The advantages of surgical interventions are the elimination of the source of emboli, removal of the painful mass, relief of ulnar nerve compression, and local periarterial sympathectomy [11]. If there is unsalvageable digital ischemia, amputation of digits is needed.

## Conclusions

UAA is a rare cause of digital ischemia, often resulting from repetitive trauma. It can be treated with low morbidity if identified early. Therefore, awareness of the condition and detailed patient assessment are needed. Early surgical intervention is the preferred primary treatment.
